# NACs strike again: NOR-like1 is responsible for cuticle development in tomato fruit

**DOI:** 10.1093/jxb/erae049

**Published:** 2024-03-27

**Authors:** Nigel E Gapper

**Affiliations:** The New Zealand Institute for Plant and Food Research Limited, Mount Albert Research Centre, Auckland, New Zealand

**Keywords:** Cuticle, cuticular wax, cutin, fruit development, NAC transcription factors, tomato

## Abstract

This article comments on:

Liu G-S, Huang H, Grierson D, Gao Y, Ji X, Peng Z-Z, Li H-L, Niu X-L, Jia W, He J-L, Xiang L-T, Gao H-Y, Qu G-Q, Zhu H-L, Zhu B-Z, Luo Y-B, Fu D-Q. 2024. NAC transcription factor SlNOR-like1 plays a dual regulatory role in tomato fruit cuticle formation. Journal of Experimental Botany 75, 1903–1918.

**NAC domain proteins are plant-specific transcription factors (TFs) that have diverged into one of the largest TF families (** **Mathew and Agarwal, 2018** **). They are critical regulators of many aspects of plant stress response and growth, including fruit development, maturation, and ripening, yet many of the NAC TFs encoded in sequenced plant genomes have not been functionally characterized. Understanding the function of these ‘orphan’ NAC TFs in crop plants is important to improve the genetic background of crops, or horticultural practices. In this issue Liu *et al.* (2024) describe an additional function for the NAC TF SlNOR-like1 that was previously reported to positively regulate fruit ripening and negatively regulate fruit size in tomato (** **Gao *et al.*, 2018** **). This new research demonstrates a dual function for SlNOR-like1 in cuticle development, through promotion of cutin deposition by activating the transcription of cutin synthesis genes, resulting in cuticle thickening in the outer layer of fruit, and also inhibition of wax accumulation by acting as a transcriptional repressor to key wax biosynthesis- and transport-related genes. These new findings provide a new model for cuticle development in fruit, an important trait that traditional plant breeding and biotechnology approaches can target.**

## The cuticle is important for fruit quality

Fruit are an essential nutritional component of the human diet and are an integral part of the world economy. Consequently, a clear understanding of fruit development is critical for optimization of fruit quality, yield, and post-harvest storage. Fruit skin composition varies widely among species. Most fleshy fruit have a live skin enveloped by a cuticle comprising cutin, a stable and insoluble polyester of primarily long chain hydroxy fatty acids (Fich *et al.*, 2016; Macnee *et al.*, 2021). The cuticle can also contain hydrophobic compounds collectively referred to as waxes, as well as cell wall polysaccharides and sometimes a saponification-resistant fraction called cutan (Nip *et al.*, 1986). The specific make up of this cuticle layer is dependent on the organ and species of origin, and serves three main functions: (i) a barrier to prevent water loss to aerial plant organs; (ii) a physical barrier to prevent pathogen infection and (iii) organogenesis and a matrix for biomechanical stability, for example to inhibit fruit cracking (Fich *et al.*, 2016). From a post-harvest perspective, a well-developed cuticle is essential for managing water loss, which is a key driver of fruit softening, for example in apples and other fruit during storage. Soft apples are not only undesirable for eating, but also susceptible to both physiological storage disorders and opportunistic pathogenic diseases. Hence, there is growing interest in post-harvest research into cuticle development and how this new knowledge can be applied in our fruit supply chains.

## Cuticle development in fruit

Although the structure of cutin polymers is still not well understood, the biosynthesis of the cuticle in plants is well established, mainly through genetic studies in Arabidopsis (Fich *et al.*, 2016; Lara *et al.*, 2019). Most fruit-focused work on cuticle deposition and development has been done in tomato (López-Casado *et al.*, 2007; Girard *et al.*, 2012; Petit *et al.*, 2016; Segado *et al.*, 2020; Reynoud *et al.*, 2022). However, there has been some wider interest in other important fruiting crops given its biological importance in fruit quality maintenance. In citrus, it was found that cutin was formed before wax accumulation during cell expansion, providing the scaffold for wax assembly during fruit maturation (Wang *et al.*, 2016). Additionally, Wang *et al*. (2016) observed that a MYB TF, GL1-like, and abscisic acid (ABA) were predicted to regulate wax synthesis during fruit development. In apple, a well-developed cuticle inhibits microcracking of the epidermis which can result in disorders such as russeting or susceptibility to fungal infections such as rust (Zhang *et al.*, 2020). Furthermore, an underdeveloped cuticle can lead to a number of age-related physiological disorders. Structural studies of the apple cuticle showed that the incorporation of [^14^C]oleic acid (C18) was significantly higher than that of [^14^C]palmitic acid (C16), and the incorporation in the cutin fraction exceeded that in the wax fraction (Si *et al.*, 2021). Moreover, the make-up of the cuticle can vary markedly depending on the variety (Leide *et al.*, 2018). To better understand russeting of fruit, microscopy, transcriptomics, and targeted metabolomic profiling were used, focusing on triterpene chemistry in russeted and non-russeted closely related ‘Golden Delicious’ clones (Falginella *et al.*, 2021). These authors showed that a MYB TF, MYB66, was able to bind the promoter of the oxidosqualene cyclase OSC5, to drive the production of lupeol derivatives. Their results provide insights into the breakdown of cuticle integrity leading to russet and how this drives MYB-regulated changes to triterpene biosynthesis. A genetic study in kiwifruit revealed three separate quantitative trait loci (QTLs) responsible for the russeting phenotype (Macnee *et al.*, 2021), highlighting the polygenic nature of cuticle development regulation in fruit.

Box 1. **Plant life cycle regulated by NAC transcription factors**

**Fig. 1.**
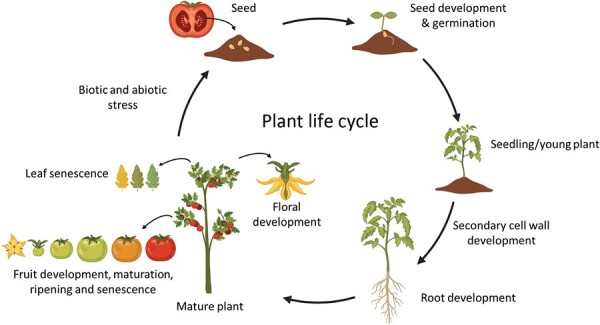
NAC TFs have been shown to regulate key aspects of plant growth, development, and stress response throughout a plant’s life cycle (tomato depicted as an example), see Box 1 for details and references. BioRender was used for graphics (https://www.biorender.com).

The sequenced tomato genome (Sato *et al.*, 2012) provides a resource for predicting and identifying gene families in a crop that produces fleshy fruit, something that the plant model Arabidopsis lacks. Bioinformatic analysis of the tomato genome has revealed as many as 104 SlNACs mapped to all 12 chromosomes (Sakuraba *et al.*, 2015). Yet many of these SlNACs are not yet functionally characterized. The life cycle of plants and diversity of regulatory function of the NAC TFs in tomato and other species is depicted in Fig. 1. The following processes are all regulated by NAC TFs: seed development and germination (Verza *et al.*, 2011; Wang *et al.*, 2014; Mathew *et al.*, 2016; Song *et al.*, 2022); secondary cell wall development (Hussey *et al.*, 2011; Chai *et al.*, 2015; Matias Hurtado *et al.*, 2020; Sun *et al.*, 2020); root development (Montiel *et al.*, 2004; Zhang *et al.*, 2018; Xu *et al.*, 2022); floral development (Sablowski and Meyerowitz, 1998; Yoo *et al.*, 2007; Zhong *et al.*, 2016; Zong *et al.*, 2020); fruit development, maturation, and ripening (for recent review articles, see Forlani *et al*., 2021; Kou *et al*., 2021; Liu *et al*., 2022, 2023); leaf senescence (Guo and Gan, 2006; Yang *et al.*, 2014; Fan *et al.*, 2015; Ma *et al.*, 2018); temperature stress (Zhou *et al.*, 2011; Guan *et al.*, 2014; Liu *et al.*, 2019; Yong *et al.*, 2019); drought stress (Sosa-Valencia *et al.*, 2017; Selote *et al.*, 2018; Sakuraba *et al.*, 2020); and biotic stress (Shan *et al.*, 2016; Guo *et al.*, 2017; Wang *et al.*, 2020; Li *et al.*, 2021).

## NAC TFs regulate fruit development, maturation, and ripening

It is well documented that fruit development, maturation, and ripening are regulated by NAC TFs (for recent review articles, see Forlani *et al.*, 2021; Kou *et al.*, 2021; Liu *et al.*, 2022, 2023). NAC-NOR, the NAC TF encoded by the *non-ripening* (*nor*) mutant in tomato, is the longest studied NAC TF involved in fruit ripening. It regulates multiple ripening pathways by activating the transcription of *SlACS2*, *SlGgpps2*, and *SlPL*, which are involved in ethylene biosynthesis, carotenoid production, and fruit softening, respectively (Gao *et al.*, 2020). In addition, NAC-NOR binds to the promoter of *SlDML2*, a DNA demethylase-encoding gene, activating its expression to enhance fruit ripening (Gao *et al.*, 2022). SlDML2 demethylates the promoter regions of important ripening genes, allowing TFs such as NAC-NOR to bind and activate transcription. NAC-NOR is also a target itself of this demethylation (Zhong *et al.*, 2013). Further, SlNAM1 accelerates ripening when overexpressed, and delays the process in CRISPR/Cas9 [clustered regularly interspaced palindromic repeats (CRISPR)/CRISPR-associated protein9]-generated mutants (Gao *et al.*, 2021). These authors also observed that SlNAM1 promoted ethylene biosynthesis through activation of *SlACS2* and *SlACS4*, the two main ACC synthase genes expressed in tomato fruit during ripening.

A third NAC TF has also been found to regulate fruit ripening in tomato, SlNOR-like1, which acts similarly to NAC-NOR by activating multiple aspects of fruit ripening (Gao *et al.*, 2018). In addition, SlNOR-like1 can control fruit size by negatively regulating cell layer number and cell area (Peng *et al.*, 2023). Liu *et al.* (2024) observed that these CRISPR/Cas9-generated mutant lines (*nor-like1*) had a micro-cracking phenotype which prompted a more in-depth study of these lines. They found that the micro-cracking phenotype resulted from reduced cutin deposition and cuticle thickness as well as increased wax accumulation. SlNOR-like1 binds to the promoter of *glycerol-3-phosphate acyltransferase6* (*SlGPAT6*), a key step in cutin monomer production, and *CUTIN DEFICIENT2* (*SlCD2*) a positive regulator of cutin production. On binding SlNOR-like1, these genes are transcriptionally activated. Conversely, the authors of this study found that SlNOR-like1 acts as a transcriptional repressor for wax biosynthesis- and transport-related genes *3-ketoacyl-CoA synthase1* (*SlKCS1*), *ECERIFERUM 1–2* (*SlCER1–2*), *SlWAX2*, and *glycosylphosphatidylinositol-anchored lipid transfer protein1-like* (*SlLTPG1-like*). These findings illustrate yet another important role for NAC TFs in plant growth and development, and provide a new model for the transcriptional regulation of the fruit cuticle.

The biological significance of cuticle development in fruit is far reaching, and these novel findings therefore provide new targets for biotechnological approaches to improve fruit quality and storability, accelerating traditional breeding efforts or through gene editing approaches. Moreover, these results illustrate the importance of the continued efforts to characterize the remaining ‘orphan’ NAC TF family members to assign biological function. Undoubtedly, there is plenty of biology left to discover from this important class of regulatory TFs.
